# Adherence to the Mediterranean Diet and Its Influence on Anthropometric and Fitness Variables in High-Level Adolescent Athletes

**DOI:** 10.3390/nu16050624

**Published:** 2024-02-23

**Authors:** Antonio E. Vélez-Alcázar, Juan Alfonso García-Roca, Raquel Vaquero-Cristóbal

**Affiliations:** 1 Facultad de Deporte, UCAM Universidad Católica de Murcia, 30310 Cartagena, Spain; aevelez@alu.ucam.edu; 2 Olympics Studies Center, UCAM Universidad Católica de Murcia, 30310 Cartagena, Spain; 3 Department of Physical Activity and Sport, Faculty of Sport Sciences, University of Murcia, 30720 Murcia, Spain

**Keywords:** mediterranean diet, athlete, adherence, anthropometry, physical fitness

## Abstract

The objectives of the present research were to analyze adherence to the Mediterranean diet (AMD), to observe which variables most affect AMD, and to analyze whether AMD affects physical fitness and anthropometric parameters in high-level adolescent athletes. A total of 96 adolescent athletes in the under-16, under-18, and under-20 categories selected by the Athletics Federation of the Region of Murcia, of whom 47 were male (age = 18.31 ± 2.31 years old) and 49 female (age = 17.27 ± 1.44 years old), participated in this study. They completed the KIDMED questionnaire to discover their AMD, as well as an anthropometric and physical condition assessment. Results: The findings show that 61.45% had an excellent degree of AMD, 31.25% a moderate one, and 7.30% a poor one. The parameters that most conditioned AMD were the consumption of fruit, vegetables, nuts, legumes, fish, breakfast cereals, and dairy products (*p* = 0.011–0.000). AMD did not show significant differences in anthropometric characteristics and physical fitness (*p* = 0.057–0.996). Conclusions: The majority of high-level adolescent athletes have a moderate or excellent AMD. The degree of AMD seems to have no influence on physical fitness and anthropometric parameters in this population.

## 1. Introduction

Physical activity during adolescence has been shown to provide numerous benefits for the prevention and treatment of chronic diseases [1] including obesity [2], hypertension [3], diabetes [4], and metabolic syndrome [5]. Longitudinal studies have shown that moderate- to vigorous-intensity physical activity (MVPA) is positively associated with increased bone mineral density [6], good cardiorespiratory fitness [7], lower blood pressure [8], a lipid profile characterized by lower total and LDL cholesterol and higher HDL [9], and improved insulin sensitivity [9], all of which are protective factors against chronic diseases [10]. Despite the benefits of physical activity from an early age, it has previously been found that during pre-adolescence and adolescence, there is a decrease in the practice of MVPA, at a rate of 3.4% in males and 5.3% in females per year from the age of 9 years [11]. This results in adolescents underperforming in health-related physical fitness parameters [12], both during this stage and in future stages [13]. More specifically, the components of physical fitness, such as cardiorespiratory capacity, strength, flexibility, and body composition, have been most closely related to good health [14], and these aspects are directly related to the maintenance of an active lifestyle [15].

Another of the issues most closely related to healthy lifestyle habits and the prevention of chronic diseases in the short, medium, and long terms in the growing-up stage is dietary habits [16]. More specifically, Mediterranean countries have been associated with specific eating habits, known as the Mediterranean diet (MD), which is characterized by a high intake of fresh foods such as fruit and vegetables, legumes, and fish, the use of olive oil, or the inclusion of cereals, among other aspects [17]. The MD is considered one of the best dietary approaches for active people and a greater AMD has been associated with a greater tendency to be physically fit [18]. Several studies generally reported higher levels of AMD in athletes with respect to inactive subjects and the general population [18]. However, in recent years, it has been found that adolescents in Mediterranean countries have also abandoned these healthy eating habits [19,20], especially those related to the consumption of fruit, vegetables, legumes, nuts, and fresh fish, adopting less healthy eating patterns characterized by a higher intake of ultra-processed or fast foods [16,21]. These changes in adolescents’ adherence to the MD (AMD) have been widely associated with less healthy physical fitness and body composition [19].

There is some controversy regarding the association between different healthy habits. In some cases, it has been suggested that subjects who are more likely to be active also tend to have a greater AMD, which would maximize the positive effects of their habits on a healthy physical condition and the prevention of chronic diseases [16]. However, other studies suggest a very different tendency, in that active subjects believe that the practice of physical exercise systematically compensates for their poor eating habits, even encouraging unhealthier habits than inactive subjects [22]. These dietary habits would detract from some of the beneficial effects that may be provided by the combination of systematic physical exercise and AMD on the health and performance of adolescents. It would therefore be advisable for adolescents to both adhere to regular physical exercise and healthy dietary habits, which could lead to a healthier physical condition, healthy parameters, and higher athletic performance [23].

Along these lines, different studies have analyzed the eating habits of young athletes in different sports disciplines, both individual [22,24,25] and collective [26,27], finding that even athletes have unhealthy eating habits, as they usually believe that it is enough to adjust their eating patterns in periods closest to training and competition [28]. Other studies have found that athletes’ eating habits were higher in terms of fat intake, but despite this, they maintained a normal body composition and blood lipid profile values [29]. However, the majority of these studies have found that in general, young athletes had moderate or excellent AMD, with no relationship between the levels of AMD and the anthropometric characteristics related to the body composition of the athletes [23,28,29,30]. However, despite the fact that athletics is one of the sports with the longest tradition in Olympism and with the greatest number of medals at stake in the Olympic Games [31], and that it is considered one of the sports with the largest number of athletes dedicated to its practice in Spain [32], no studies have analyzed the relationship between AMD and athletes’ anthropometric variables, body composition, and healthy physical condition.

Therefore, the objectives of this research were (a) to analyze the degree of AMD presented by adolescent athletes; (b) to observe which variables most affect AMD in young athletes; and (c) to analyze whether AMD affects anthropometric variables, body composition, and physical fitness, as well as to analyze the influence of sex on this issue.

The hypotheses of the present research were as follows: (a) athletes would mostly have a moderate or excellent AMD; (b) the variables for which athletes would present a greater discrepancy with the AMD would be those referring to the consumption of fruits, vegetables, legumes, nuts, and fish; (c) there would not be an influence of the AMD on health-related physical fitness and anthropometric parameters for the overall sample or according to sex.

## 2. Materials and Methods

### 2.1. Study Design

The present research was carried out using a cross-sectional descriptive–correlational design, in which AMD was evaluated as an independent variable and the specific items that characterize AMD and anthropometry variables, body composition, and physical fitness (strength; flexibility; and velocity) as dependent variables. Data collection took place in November 2022.

Before starting this study, the institutional ethics committee of the Catholic University of Murcia approved the protocol designed for the research study (protocol code: CE 052303), according to the Helsinki declaration. The STROBE statement was followed for the development of the manuscript [33]. Coaches, parents, and athletes were informed of the measurement protocol, and the athletes signed an informed consent form before starting the study; the same form was signed by the parents of underage athletes.

The sample was selected using a non-probabilistic method of coexistence among athletes belonging to the Athletics Federation of the Region of Murcia (FAMU), an autonomous community located in the southeast of Spain. The selection criteria were to be among the best in their category and modality and/or competing in the First National Athletics Division.

Rstudio software (version 3.15.0, Rstudio Inc., Boston, MA, USA) was used for sample size calculations. The significance level was set a priori at α = 0.05. The standard deviation was set according to previous studies based on AMD in adolescents (SD = 2.32) [34]. The minimum sample size of the present investigation was 96 athletes considering an estimated error (d) of 0.46 for a 95% confidence interval.

### 2.2. Participants

The sample comprised a total of 96 under-16, under-18, and under-20 athletes selected by the FAMU for being the best in their category and modality and/or competing in the First National Athletics Division, of whom 47 were male (age = 18.31 ± 2.31 years old) and 49 were female (age = 17.27 ± 1.44 years old). The inclusion criteria were (a) to be federated in athletics in the FAMU for at least one season; (b) belonging to the U-16, U-18, or U-20 categories; (c) practicing one of the following athletics disciplines: running, jumping, throwing, or combined track and field events; and (d) to have trained a minimum of three days a week, accumulating a minimum of four hours of training per week during the last year. The exclusion criteria for the study were (a) having suffered an injury in the last three months that prevented them from training or competing normally; (b) not completing any of the tests foreseen in the protocol; (c) having suffered any illness that prevented them from training or competing normally in the last three months; (d) having missed more than 20% of training sessions in the last month; and (e) practicing another sport as a federated athlete.

### 2.3. Measurements

#### 2.3.1. Questionnaire Measurements

In order to collect information on socio-demographic and athletics-related aspects, the athletes completed an ad hoc questionnaire asking about age and sex; years of experience as federated athletes; days and hours per week they had spent training in athletics in the last year; whether they had suffered an injury in the last three months that had prevented them from training or competing normally; whether they had suffered from any other incapacitating illness in the last three months that had prevented them from training or competing normally; if they practiced any other sport as a federated athlete; and how many training sessions they had missed in the last month.

For the assessment of diet and AMD, athletes self-completed the KIDMED questionnaire [16]. The development of the KIDMED questionnaire is based on the principles underlying the Mediterranean dietary patterns. This questionnaire has been validated to assess adolescents’ AMD [16,35]. In fact, it has been a commonly used questionnaire to assess AMD in trainee athletes [27]. The questionnaire consists of 16 dichotomous response questions (“yes”, “no”), with scores ranging from 0 to 1 point for items related to increased AMD, and −1 to 0 points for items that ask about the negative aspects of AMD [16]. The final score of the questionnaire ranged from 0 to 12 points, and subjects were classified into three levels according to their final score: excellent AMD (>8 points); moderate AMD (4–7 points); and poor AMD (<3 points).

#### 2.3.2. Anthropometric Measurements

Subjects underwent an anthropometric assessment, following the guidelines from the International Society for the Advancement in Kinanthropometry (ISAK) [36]. All measurements were taken by two level 3 and 4 anthropometrists with current ISAK accreditation. The number of measurements performed for each variable was in accordance with ISAK guidelines [36,37].

Body mass measurements were taken with an SECA 862 scale with a precision of 100 g (SECA, Hamburg, Germany); height and sitting height with a stadiometer with a precision of 1 mm (SECA, Germany); triceps, thigh, and calf skinfolds with a caliper with a precision of 0.2 mm (Harpenden, Harpenden, UK); relaxed arm, thigh middle, and leg girths with an inextensible tape with a precision of 1 mm (Lufkin, Missouri City, TX, USA); and humerus, bi-styloid, and femur breadths with a small sliding caliper with a precision of 1 mm (Holtain, Crymych, UK). The intra-evaluator TEM was 0.03% for basic measurements, 2.24% for skinfolds, 0.36% for girths, and 0.48% for breadths, and its correlation coefficient with an expert level 4 anthropometrist was 0.99 for basic measurements, 0.91 for skinfolds, 0.98 for girths, and 0.96 for breadths. The temperature of the room where the measurements were taken was standardized at 24 °C, and all measurements were taken from 9:00 to 14:00 on two consecutive days.

The final values of the anthropometric measurements were used to calculate the body mass index (BMI) [37]; Σ3 skinfolds [37]; fat mass [38], muscle mass [39], and bone mass in kg and percentage [40]; muscle–bone index [41]; corrected arm girth, corrected thigh girth, and corrected leg girth [37]; and Σ3 corrected girths [37].

The sex-specific formula [42] was used to estimate the age at peak height velocity (APHV) maturity offset in years. This method has been shown to be valid for estimating maturational lag with respect to the gold standard (left wrist radiograph), with an R^2^ = 0.92–0.89 for males and an R^2^ = 0.91–0.88 for females [42].

#### 2.3.3. Physical Fitness Tests

Familiarization and assessment of the fitness tests were carried out by four researchers with previous experience in the field. Each researcher supervised the same fitness tests during all the measurements to avoid inter-rater errors.

The sit-and-reach test was assessed with an ACUFLEX TESTER III box (Novel Products, Pittsburgh, PA, USA) following the methodology of previous research [43,44]. The distance achieved was recorded in cm. A single attempt of the test was performed, following previous research [43,44].

For the CMJ and SJ tests, a force platform with a sampling frequency of 200 Hz was used (MuscleLab, Stathelle, Norway). The CMJ test consisted of the execution of a maximum counter movement vertical jump, following the protocol of previous studies [45]. For the SJ test, the subject performed a maximum vertical jump, without counter movement, from a semi-squat position [46]. In both CMJ and SJ, the arms were placed on the hips to avoid their influence on the test result [47]. For the horizontal jump test, athletes had to perform a maximum forward jump, with both feet at the same time, trying to reach the maximum possible distance, following the methodology of previous studies. The use of the arms was allowed in the push to maintain balance [48]. Two non-consecutive trials of each jump test were performed, with the final value being the maximum value of both attempts, following the methodology from previous research [48]. The distance achieved was recorded in cm for all the jump tests.

The handgrip test was performed with a Takei Tkk5401 digital dynamometer (Takei Scientific Instruments, Niigata-shi, Niigata Prefecture, Japan). Participants were asked to squeeze the dynamometer handle as hard as possible. They performed the test alternately with both arms [49]. The value was recorded in kg. To perform the medicine ball test, athletes had to throw a three-kilogram medicine ball (Technogym, Cesena, Italy) with both arms over the head, following the methodology of previous studies [50]. The distance was measured in m. Two attempts were performed for both upper strength tests in a non-consecutive manner, with the final value being the maximum of both attempts, following the methodology from previous research [51].

With respect to the 30 m sprint test, two pairs of photoelectric cells (Microgate, Bolzano, Italy) were used to record the time taken by the athlete to cover this distance. Athletes started from a squat position (three supports), following previous research [52]. The time was recorded in s. When the test was performed outdoors, wind speed was controlled by placing an anemometer Gill-compact (Gill Athletics, Champaign, IL, USA) halfway along the course, recording tests that were below 2 m per second in the direction of the run [53]. Two attempts were performed, with the final value being the maximum of both attempts, following the methodology from previous research [27].

### 2.4. Protocol

The tests were carried out in the morning on two consecutive days at the municipal athletics track in Cartagena, to reduce the possible contaminating variables that could affect the results.

In the first instance, all athletes self-completed the socio-demographic questionnaire (ad hoc) and the AMD questionnaire (KIDMED test). This was followed by the assessment of anthropometric variables. Subsequently, the sit-and-reach test was performed to assess hamstring flexibility. This was performed before any type of warm-up, to avoid the immediate effect of the warm-up on hamstring extensibility values [54]. A standardized warm-up was then performed, consisting of 5 min of continuous running, followed by joint mobility exercises. After this, the correct execution of the physical fitness tests was explained to the athletes, and a familiarization session was carried out for the tests that could be influenced by the execution technique (vertical jump -CMJ and SJ-, horizontal jump, and medicine ball throw), but without asking the subjects to perform a maximum attempt to reduce the appearance of fatigue. After this familiarization session, to finalize the warm-up, five minutes of progressive sprints (40 m at 50%, 70%, and 90% effort) was performed [52]. At the end of this warm-up, the CMJ, SJ, horizontal jump, medicine ball throw, handgrip, and 30 m sprint tests were performed in a random order. Two minutes of recovery time was allowed between the two measurements of each test and five minutes between the different tests. The test protocol was established according to the guidelines written by the National Strength and Conditioning Association (NSCA) [10]. These recommended guidelines assess the fatigue generated by each test and establish a recovery time interval to mitigate possible interferences [55].

### 2.5. Statistical Analysis

The normality, homogeneity, and sphericity of the data were evaluated using the Kolmogorov–Smirnov, Levene, and Mauchly tests, respectively. Since all the variables analyzed showed a normal distribution, parametric tests were used. Descriptive statistics, such as mean values and standard deviation, were utilized to analyze quantitative variables, whereas qualitative variables were analyzed using frequencies and percentages. A one-way ANCOVA was performed to compare the differences between the AMD groups in the anthropometric variables, body composition, and physical fitness tests and to measure the influence of sex on these differences. Both the main effects and the interaction between the variables were analyzed. The effect size was calculated with partial eta squared (η^2^). The Bonferroni post hoc adjustment was used to analyze differences between groups when these differences were significant. 

A chi-square analysis (χ^2^) made it possible to establish the differences in the KIDMED items between groups according to their AMD. Cramer’s V was used for the post hoc comparison of the 2 × 2 tables, and the contingency coefficient was used in the 2 × n tables, to obtain the statistical value. The maximum expected value was 0.707; r < 0.3 indicated a poor association; r < 0.5 indicated a moderate association; and r > 0.5 indicated a high association [56]. A *p* < 0.05 value was set to determine statistical significance. The statistical analysis was performed using the SPSS statistical package (v.25.0; SPSS Inc., Chicago, IL, USA).

## 3. Results

The socio-demographic characteristics of the sample are given in Table 1.

The sample of 96 young athletes was divided into three groups according to their degree of AMD. It was observed that 59 athletes (61.45%) showed an excellent degree of AMD, 30 athletes (31.25%) showed a moderate degree of AMD, and 7 athletes (7.30%) showed a poor degree of AMD.

Table 2 shows the differences in terms of AMD for the different items assessed. It was found that, within the poor AMD group, there was a significantly higher percentage of subjects who did not consume a piece of fruit or natural juice every day, who did not consume a second piece of fruit a day, who did not eat fresh vegetables once a day, who did not eat a second unit of fresh vegetables a day, who did not eat fish regularly, who did not eat cereal or derivatives or a dairy product for breakfast, who did not eat nuts regularly, who did not use olive oil at home, and who did not eat two yogurts and/or 40 g of cheese per day. On the other hand, most of them ate breakfast regularly (*p* = 0.001–0.000).

In the moderate AMD group, there were significantly higher percentages of subjects who did not consume a piece of fruit or fresh juice every day, who did not eat fresh vegetables more than once a day, who did not eat fish regularly, who did not like legumes and consume them more than once a week, who did not eat nuts regularly, and who did not eat 40 g of cheese and/or two yogurts a day (*p* = 0.011–0.000).

In the excellent AMD group, a significantly higher percentage of subjects were found to eat one piece of fruit or drink natural juice every day, consume a second piece of fruit every day, eat one or more than one fresh vegetable daily, consume fish regularly, consume nuts regularly, like legumes and consume them more than once a week, eat a cereal/derivative and/or a dairy product for breakfast, use olive oil at home, and eat two yogurts and/or 40 g of cheese daily. In contrast, it was found that some of the subjects with an excellent AMD did not eat breakfast (*p* = 0.011–0.000).

Table 3 shows the descriptive data (mean ± SD) for anthropometric characteristics and physical fitness as a function of the degree of AMD, as well as the effect of the covariate sex on the differences between groups. For the general model, no significant differences were found in any of the variables analyzed (*p* = 0.057–0.996). On the other hand, when introducing the sex covariate, significant differences were found between groups in the variables body mass (kg) (*p* = 0.008); bone mass (kg) (*p* = 0.000); muscle mass (kg) (*p* = 0.000); ∑3 skinfolds (mm) (*p* = 0.000); ∑corrected girths (cm) (*p* = 0.000); fat mass (%) (*p* = 0.000); adipose mass (%) (*p* = 0.000); bone mass (%) (*p* = 0.006); muscle–bone index (*p* = 0.003); handgrip right (kg) (*p* = 0.000); handgrip left (kg) (*p* = 0.000); CMJ (cm) (*p* = 0.000); SJ (cm) (*p* = 0.000); horizontal jump test (cm) (*p* = 0.000); medicine ball throw (m) (*p* = 0.000); and 30 m sprint (s) (*p* = 0.000).

Table 4 shows the Bonferroni adjustment for those anthropometric variables and physical fitness variables for which significant differences were found after the ANCOVA according to the degree of AMD. No significant differences were found between groups in any of the variables analyzed after the adjustment (*p* = 0.204–1.000).

## 4. Discussion

The first objective of this research was to analyze the degree of AMD in adolescent athletes. It was found that 61.45% of the athletes showed an excellent degree of AMD, while 31.25% of the athletes showed a moderate one. The data of the present research, in which high rates of AMD were found, contrast with those found in the young population, where a lower AMD has been found in recent years due to their aversion to the consumption of different types of food and to the fact that their consumption is predominantly driven by their food preferences [57,58,59], which leads to an increasing prevalence of ultra-processed and fast food consumption in this population [16,21,60]. It should be added that AMD at this early age may depend on other issues such as the cultural eating habits of the parents and family [35]. In this respect, the total sample of the present investigation were Spanish Caucasian athletes, so similar acquired eating habits are assumed [35,61]. However, this is undoubtedly an issue to be analyzed in the future in the case of heterogeneous samples in terms of cultural eating habits.

The trend found in the present study on the grade of AMD is along the same lines as previous studies focused on the athlete population. Thus, in elite canoeists [29] and adolescent kayakers and canoeists [28,30], excellent and moderate AMD were found [28,29,30]. Similarly, research on adolescents and young football and handball players found a major tendency to have a moderate and/or excellent AMD [41,62,63]. In another study, it was found that the major tendency in young beach handball players was to have a moderate AMD [64]. Therefore, in general, there is a tendency for the athlete population to have a moderate or excellent degree of AMD. The differences between the different studies, with respect to the percentage of young athletes who have an excellent or moderate AMD, may be due to the heterogeneity of the samples analyzed, the degree of professionalization of the different sports, and the competitive level of the participants [30]. Based on these results, the initial hypothesis can be accepted, confirming that the majority of high-level athletes have excellent or moderate AMD.

The second objective of this research was to discover which variables most affect AMD in young athletes. When analyzing the consumption of each of the foods, it was found that the majority consumed a piece of fruit or natural juice, vegetables, dairy products, and olive oil every day; a second piece of fruit and vegetables every day; and nuts, legumes, and fish on a regular basis. However, the degree of compliance with the consumption of these products depended on the degree of AMD, with excellent adherence being key to meeting the recommended consumption within the parameters of the MD. Previous studies have pointed to the consumption of fruit, vegetables, nuts, legumes, and fish as one aspect in which individuals were the most non-compliant in terms of AMD, as the consumption of most of these products tends to generate aversion among young people, who adjust their nutritional habits to their culinary preferences [28,58,60]. The intake of these food groups is important, as they contain bioactive compounds such as antioxidants, fiber, and phytosterols; monounsaturated fatty acids and omega-3 fatty acids; and probiotics [65]. These compounds could benefit the health and performance status of athletes by attenuating the production of oxidative stress, thereby improving muscle performance and immune function [66], and by affecting satiety, which could contribute to body weight control [67]. Therefore, monitoring the consumption of these food groups is one of the key issues to review in the compliance of athletes in training with the MD.

In the present investigation, it was found that a large majority of athletes consumed cereals or derivatives and dairy products during breakfast, with differences in these items depending on the degree of AMD, where athletes with an excellent degree of adherence showed a higher compliance with these items. Following this line, a previous study found that athletes consumed dairy products and cereals in combination during breakfast [64]. Previous studies indicate that the consumption of cereals has health benefits, such as improved intestinal flora, better regulation of plasma glucose, and prevention of cardiovascular or chronic metabolic diseases [68]. However, a surprising result of the present investigation was that although a large majority of the athletes ate breakfast, the percentage of those who did not comply with this habit was higher among those with an excellent AMD. Breakfast is the key meal of the day, as it covers 25% of the daily nutritional needs. Therefore, not eating breakfast or having an insufficient/poor breakfast can affect the physical and intellectual activity of an individual [69]. Therefore, as a practical application of this, it would be necessary to check which athletes eat breakfast and what they include in their breakfasts on a regular basis.

Finally, a relevant result of the present research was that a large majority of athletes did not regularly visit fast-food restaurants, did not eat industrial pastries for breakfast, and did not eat sweets and/or candies, with no differences in these items depending on the degree of AMD. Sugary breakfast cereals, bakery products, snacks, high-fat savory foods, soft drinks, and fast-food restaurants are the foods with the highest percentage of advertising [70], and yet the participants in the present research showed low consumption of these types of foods, which could be due to the self-regulatory behaviors among such athletes [71]. Based on the above, the second hypothesis can be partially accepted, as the intake of the fruit, vegetable, legume, nut, and fish food groups showed the greatest discrepancy in their consumption according to the degree of AMD, just as the consumption of dairy products and the habit of eating breakfast in the morning. Based on these results, special attention should be paid to certain food groups when monitoring the dietary habits of the athlete population.

The third objective of this research was to analyze whether AMD affects anthropometric variables, body composition, and physical fitness, and to analyze the influence of sex on this issue. With respect to the results obtained, no statistically significant differences were found in physical fitness, anthropometric parameters, and body composition as a function of the degree of AMD. These results coincide with those found in previous studies on adolescents and young athletes in both individual and team sports [30,62,64]. No significant differences were found according to sex, in line with previous research in the growing population [72,73]. Therefore, having a moderate or excellent AMD does not seem to be a determinant factor for better results in different fitness levels, anthropometric parameters, and body compositions in adolescents and young athletes [64]. These findings could be due to the homogeneity of AMD due to the sample analyzed, as has been pointed out in previous studies [30]. However, more related research with a larger sample size would be needed to further analyze whether an excellent or poor AMD influences fitness tests or anthropometric values.

Therefore, the third hypothesis of the present research can be confirmed, as there seems to be no influence of the AMD on physical fitness, anthropometric parameters, and body composition for the overall sample, and neither according to sex. In view of the above, having an excellent AMD would not by itself be a sufficient parameter to explain the differences in the anthropometric, body composition, and physical fitness parameters of high-level athletes in training.

As possible practical applications of the present study, this is the first research study to present data on the degree of AMD of high-level athletes in training, the food groups that show the greatest differences in terms of the degree of adherence, and the influence of these aspects on anthropometric variables, body composition, and physical condition. The results shown in this study can be used to define nutritional guidelines as advised and guided by professionals, with the aim of individualizing the nutritional plans for competition and training, to optimize sports performance and health-related parameters in young athletes [74,75]. The results found can also help guide training programs provided from an early age to raise awareness of the importance of monitoring and controlling food intake in order to optimize performance and health in any type of sporting discipline [62]. Finally, the present study shows that sports practice is associated with healthy eating habits.

Regarding the limitations of this research, the main one was the sample size. Secondly, it should be noted that the KIDMED questionnaire does not detail quantities, so it was not possible to calculate the intake of macronutrients, micronutrients, or energy balance. Thirdly, as it is a cross-sectional study, nutritional interventions based on the characteristics of the MD were not carried out, which could have improved the performance, physical fitness, and anthropometric parameters of the athletes, based on the findings from previous studies [62,64,73]. So, future studies should examine these questions with prospective or longitudinal designs. Fourthly, this research did not differentiate between the different types of athletics, given the size of the sample, but analyzed athletics as a whole. Fifth, although the equations for estimating body composition used in this research were chosen because they are recommended for growing populations [76], recent research has shown that the data reported by different body composition methods and equations differ from each other [77], so the data obtained for body composition in this research would have differed if other equations had been used [77]. Finally, the present investigation did not analyze the metabolic expenditure of the athletes, which could affect the anthropometric results of the athletes [78]. This is an issue to be included in future research.

## 5. Conclusions

From the total sample, 61.45% of elite athletes in training showed an excellent degree of AMD, while 31.25% of the athletes showed a moderate degree of AMD. The items that showed the greatest differences according to the degree of AMD were the consumption of fruit, vegetables, legumes, nuts, fish, and dairy products, as well as the consumption of cereals or derivatives and dairy products at breakfast. The degree of AMD seems to have no influence on anthropometric parameters, body composition, and physical fitness in this population.

## Figures and Tables

**Table 1:** Socio-demographic characteristics of the athletes.

	Mean ± SD/*n* (%)	Min.	Max.
Age (years old)	17.59 ± 1.97	15.24	24.89
Sex	Male: *n* = 47; Female: *n* = 49		
Race	Caucasian: *n* = 96 (100%)		
Nationality	Spanish: *n* = 96 (100%)		
Category	U-16: *n* = 33 (34.3%); U-18: *n* = 33 (34.3%); U-20: *n* = 30 (31.2%)		
Years of athletics experience (years)	3.69 ± 3.56	1	15
Athletic weekly training days (days)	4.52 ± 0.89	3	7
Athletic hours of training per week (hours)	7.34 ± 3.16	6	19
Gym hours of training per week (hours)	3.82 ± 2.67	2	16

U-16: Under-16; U-18: Under-18; U-20: Under-20.

**Table 2:** Comparison according to the degree of adherence to the Mediterranean diet (AMD) of daily intake habits through the KIDMED questionnaire.

	Poor AMD			Moderate AMD			Excellent AMD			*p*	η^2^	*r*	*V*	*C*
	YES	NO	R	YES	NO	R	YES	NO	R					
Eat a piece of fruit or drink natural juice every day	3 (42.9%)	4 (57.1%)	−3.1	22 (73.3%)	8 (26.7%)	−2	56 (94.9%)	3 (5.1%)	3.6	0.000	0.417	0.000	0.000	0.000
Eat a 2nd piece of fruit every day	1 (14.3%)	6 (85.7%)	−3.1	18 (60.0%)	12 (40.0%)	−1.1	46 (78.0%)	13 (22.0%)	2.7	0.000	0.347	0.002	0.002	0.002
Eat fresh/cooked vegetables once a day	2 (28.6%)	5 (71.4%)	−3.6	21 (70.0%)	9 (30.0%)	−1.7	54 (91.5%)	5 (8.5%)	3.5	0.000	0.429	0.000	0.000	0.000
Eat fresh/cooked vegetables more than once a day	0 (0.0%)	7 (100.0%)	−2.4	6 (20.0%)	24 (80.0%)	−3.2	36 (61.0%)	23 (39.0%)	4.3	0.000	0.443	0.000	0.000	0.000
Eat fish at least 2–3 times a week	2 (28.6%)	5 (71.4%)	−2.5	15 (50.0%)	15 (50.0%)	−2.8	50 (84.7%)	9 (15.3%)	4.0	0.000	0.423	0.000	0.000	0.000
Go to a fast-food restaurant once a week or more	2 (28.6%)	5 (71.4%)	1.0	6 (20.0%)	24 (80.0%)	0.8	7 (11.9%)	52 (88.1)	−1.3	0.376	0.143	0.376	0.376	0.376
Likes legumes and consumes more than once a week	4 (57.1%)	3 (42.9%)	−1.4	19 (63.3%)	11 (36.7%)	−2.4	52 (88.1%)	7 (11.9%)	3.0	0.011	0.296	0.011	0.011	0.011
Eat pasta or rice almost every day (5 or more days)	6 (85.7%)	1 (14.3%)	1.9	11 (36.7%)	19 (63.3%)	−1.9	32 (54.2%)	27 (45.8%)	0.8	0.048	0.018	0.048	0.048	0.048
Eat a cereal or derivative (bread, etc.) for breakfast	0 (0.0%)	7 (100.0%)	−4.2	18 (60.0%)	12 (40.0%)	−1.4	49 (83.1%)	10 (16.9%)	3.6	0.000	0.459	0.000	0.000	0.000
Eat nuts (at least 2–3 times a week)	1 (14.3%)	6 (85.7%)	−2.3	10 (33.3%)	20 (66.7%)	−3.1	43 (72.9%)	16 (27.1%)	4.1	0.000	0.426	0.000	0.000	0.000
Olive oil is used at home	5 (71.4%)	2 (28.6%)	−2.9	29 (96.7%)	1 (3.3%)	0.6	57 (96.6%)	2 (3.4%)	1.0	0.015	0.202	0.015	0.015	0.015
No breakfast	4 (57.1%)	3 (42.9%)	4.9	3 (10.0%)	27 (90.0%)	0.4	1 (1.7%)	58 (98.3%)	−3.0	0.000	0.440	0.000	0.000	0.000
Have a dairy breakfast (yogurt, etc.)	3 (42.9%)	4 (57.1%)	−3.0	22 (73.3%)	8 (26.7%)	−1.8	55 (93.2%)	4 (6.8%)	3.3	0.001	0.386	0.001	0.001	0.001
Eat industrial pastries for breakfast	2 (28.6%)	5 (71.4%)	1.3	6 (20.0%)	24 (80.0%)	1.5	4 (6.8%)	55 (93.2%)	−2.1	0.084	0.226	0.084	0.084	0.084
Have 2 yogurts and/or 40 g of cheese every day	0 (0.0%)	7 (100.0%)	−2.4	3 (10.0%)	27 (90.0%)	−4.4	38 (64.4%)	21 (35.6%)	5.4	0.000	0.530	0.000	0.000	0.000
Eat sweets and/or candies several times a day	2 (28.6%)	5 (71.4%)	1.8	4 (13.3%)	26 (86.7%)	0.9	3 (5.1%)	56 (94.9%)	−1.8	0.088	0.221	0.088	0.088	0.088

AMD: adherence to the Mediterranean diet.

**Table 3:** Differences in anthropometric variables and physical fitness according to the degree of adherence to the Mediterranean diet, as well as the influence of the covariate sex.

	Poor AMD	Moderate AMD	Excellent AMD	ANOVA			Group × Sex		
	Mean ± SD	Mean ± SD	Mean ± SD	*F*	*p*	η^2^*_p_*	*F*	*p*	η^2^*_p_*
Age (years old)	17.11 ± 1.55	17.90 ± 2.31	17.80 ± 1.84	0.44	0.643	0.009	2.48	0.066	0.075
Years of experience in athletics (years)	3.60 ± 3.45	6.00 ± 3.40	6.80 ± 3.60	2.85	0.063	0.058	1.88	0.138	0.058
Total hours of training per week (hours)	7.00 ± 1.52	8.83 ± 2.90	8.61 ± 3.41	0.97	0.383	0.020	2.24	0.088	0.068
Body mass (kg)	57.92 ± 13.4	65.70 ± 18.20	63.90 ± 11.21	0.88	0.417	0.019	4.22	0.008	0.121
Maturity offset (years)	2.20 ± 0.96	3.00 ± 1.71	3.14 ± 1.33	1.40	0.258	0.029	1.20	0.322	0.037
BMI (kg/m^2^)	22.15 ± 4.13	22.32 ± 4.60	21.92 ± 3.40	0.11	0.893	0.002	0.07	0.973	0.002
∑3 Skinfolds (mm)	54.06 ± 40.20	33.90 ± 17.40	40.60 ± 24.60	2.17	0.120	0.045	12.61	0.000	0.291
∑Corrected girths (cm)	95.20 ± 7.30	103.70 ± 12.40	101.80 ± 9.30	2.00	0.146	0.041	13.72	0.000	0.309
Fat mass (kg)	20.00 ± 9.72	21.70 ± 7.90	21.82 ± 6.00	0.22	0.803	0.005	1.07	0.364	0.034
Fat mass (%)	21.02 ± 12.82	15.00 ± 7.30	18.09 ± 9.91	1.66	0.194	0.035	14.18	0.000	0.316
Muscle mass (kg)	23.21 ± 2.80	27.20 ± 6.50	26.51 ± 4.03	1.88	0.157	0.039	7.41	0.000	0.195
Muscle mass (%)	34.65 ± 16.30	40.35 ± 8.23	40.16 ± 8.75	1.17	0.313	0.025	0.80	0.504	0.025
Bone mass (kg)	9.20 ± 1.80	10.50 ± 1.65	10.44 ± 1.60	2.00	0.140	0.041	31.12	0.000	0.504
Bone mass (%)	16.03 ± 1.85	16.40 ± 2.07	16.50 ± 1.70	0.20	0.814	0.004	4.42	0.006	0.126
Muscle–bone index	53.40 ± 1.80	52.53 ± 1.15	52.60 ± 1.30	1.21	0.301	0.025	4.90	0.003	0.137
Handgrip right (kg)	30.60 ± 9.24	36.70 ± 10.50	34.35 ± 8.35	1.44	0.240	0.030	21.27	0.000	0.410
Handgrip left (kg)	27.20 ± 7.50	34.35 ± 10.60	33.10 ± 8.90	1.70	0.193	0.035	21.01	0.000	0.407
Sit-and-reach test (cm)	23.30 ± 11.13	20.21 ± 8.52	22.53 ± 9.30	0.72	0.487	0.015	2.76	0.046	0.083
CMJ (cm)	30.42 ± 5.25	34.93 ± 9.62	33.45 ± 7.82	0.90	0.410	0.019	14.08	0.000	0.315
SJ (cm)	27.60 ± 4.70	32.80 ± 8.42	31.24 ± 6.70	1.60	0.213	0.033	13.40	0.000	0.304
Horizontal jump test (cm)	202.90 ± 28.06	208.33 ± 32.92	205.50 ± 35.00	0.10	0.899	0.002	14.25	0.000	0.317
Medicine ball throw (m)	5.80 ± 1.80	6.60 ± 1.90	6.80 ± 1.80	1.00	0.381	0.021	16.40	0.000	0.348
30 m sprint (s)	4.72 ± 0.35	4.54 ± 0.40	4.63 ± 0.41	1.13	0.326	0.024	16.55	0.000	0.351

AMD: adherence to the Mediterranean diet. BMI: body mass index. CMJ: counter movement jump. SJ: squat jump.

**Table 4:** Post hoc comparison according to the degree of adherence to the Mediterranean diet, including the covariate sex, in the anthropometric and physical fitness variables.

Tests	Group Comparison		Mean ± SD	*p*	95% CI
Body mass (kg)	Poor AMD	Moderate AMD	−4.90 ± 5.61	1.000	−18.60 to 8.80
	Poor AMD	Excellent AMD	−4.44 ± 5.30	1.000	−17.40 to 8.50
	Moderate AMD	Excellent AMD	0.50 ± 3.00	1.000	−6.83 to 7.74
∑3 Skinfolds (mm)	Poor AMD	Moderate AMD	12.52 ± 8.90	0.480	−9.00 to 34.05
	Poor AMD	Excellent AMD	9.30 ± 8.40	0.802	−11.03 to 29.65
	Moderate AMD	Excellent AMD	−3.22 ± 4.70	1.000	−14.70 to 8.25
∑Corrected girths (cm)	Poor AMD	Moderate AMD	−5.10 ± 3.72	0.520	−14.20 to 3.96
	Poor AMD	Excellent AMD	−4.73 ± 3.51	0.543	13.30 to 3.90
	Moderate AMD	Excellent AMD	0.40 ± 2.00	1.000	−4.50 to 5.20
Fat mass (%)	Poor AMD	Moderate AMD	2.90 ± 3.40	1.000	−5.40 to 11.08
	Poor AMD	Excellent AMD	1.20 ± 3.20	1.000	−6.60 to 9.00
	Moderate AMD	Excellent AMD	−1.70 ± 1.80	1.000	−6.05 to 2.73
Muscle mass (kg)	Poor AMD	Moderate AMD	−2.80 ± 1.91	0.462	−7.40 to 1.91
	Poor AMD	Excellent AMD	−2.62 ± 1.80	0.450	−7.02 to 1.80
	Moderate AMD	Excellent AMD	0.12 ± 1.02	1.000	−2.40 to 2.60
Bone mass (kg)	Poor AMD	Moderate AMD	−0.60 ± 0.50	0.704	−1.80 to 0.62
	Poor AMD	Excellent AMD	−0.90 ± 0.50	0.204	−2.00 to 0.30
	Moderate AMD	Excellent AMD	−0.30 ± 0.30	0.915	−0.92 to 0.40
Bone mass (%)	Poor AMD	Moderate AMD	0.03 ± 0.73	1.000	−1.80 to 1.82
	Poor AMD	Excellent AMD	−0.25 ± 0.70	1.000	−2.00 to 1.50
	Moderate AMD	Excellent AMD	−0.30 ± 0.40	1.000	−1.22 to 0.70
Muscle–bone index	Poor AMD	Moderate AMD	0.60 ± 0.52	0.880	−0.72 to 1.82
	Poor AMD	Excellent AMD	0.61 ± 0.50	0.645	−0.60 to 1.82
	Moderate AMD	Excellent AMD	0.06 ± 0.30	1.000	−0.61 to 0.74
Handgrip right (kg)	Poor AMD	Moderate AMD	−2.50 ± 3.04	1.000	−10.00 to 4.90
	Poor AMD	Excellent AMD	−1.80 ± 2.90	1.000	−8.80 to 5.20
	Moderate AMD	Excellent AMD	0.70 ± 1.60	1.000	−3.25 to 4.70
Handgrip left (kg)	Poor AMD	Moderate AMD	−3.60 ± 3.20	0.800	−11.20 to 4.20
	Poor AMD	Excellent AMD	−4.00 ± −3.00	0.560	−11.20 to 3.30
	Moderate AMD	Excellent AMD	−0.41 ± 1.70	1.000	−4.50 to 3.70
CMJ (cm)	Poor AMD	Moderate AMD	−1.65 ± 3.00	1.000	−8.90 to 5.60
	Poor AMD	Excellent AMD	−1.50 ± 2.80	1.000	−8.30 to 5.40
	Moderate AMD	Excellent AMD	0.20 ± 1.60	1.000	−3.70 to 4.02
SJ (cm)	Poor AMD	Moderate AMD	−2.80 ± 2.60	0.840	−9.20 to 3.50
	Poor AMD	Excellent AMD	−2.40 ± 2.50	1.000	−8.40 to 3.70
	Moderate AMD	Excellent AMD	0.50 ± 1.40	1.000	−2.90 to 3.90
Horizontal jump test (cm)	Poor AMD	Moderate AMD	6.50 ± 12.00	1.000	−22.80 to 35.80
	Poor AMD	Excellent AMD	4.00 ± 11.33	1.000	−23.70 to 31.60
	Moderate AMD	Excellent AMD	−2.60 ± 6.40	1.000	−18.20 to 13.00
Medicine ball throw (m)	Poor AMD	Moderate AMD	−0.12 ± 0.63	1.000	−1.70 to 1.50
	Poor AMD	Excellent AMD	−0.65 ± 0.60	0.880	−2.10 to 0.82
	Moderate AMD	Excellent AMD	−0.52 ± 0.40	0.400	−1.40 to 0.30
30 m sprint (s)	Poor AMD	Moderate AMD	0.04 ± 0.20	1.000	−0.30 to 0.40
	Poor AMD	Excellent AMD	−0.02 ± 0.20	1.000	−0.40 to 0.30
	Moderate AMD	Excellent AMD	−0.06 ± 0.07	1.000	−0.30 to 0.20

AMD: adherence to the Mediterranean diet. CMJ: counter movement jump. SJ: squat jump.

## Data Availability

The data presented in this study are available on request from the corresponding author upon reasonable request.
